# Photodynamic Therapy Using a Novel Phosphorus Tetraphenylporphyrin Induces an Anticancer Effect via Bax/Bcl-xL-related Mitochondrial Apoptosis in Biliary Cancer Cells

**DOI:** 10.1267/ahc.20-00002

**Published:** 2020-07-04

**Authors:** Nguyen Nhat Huynh Mai, Yuya Yamaguchi, Narantsog Choijookhuu, Jin Matsumoto, Atsushi Nanashima, Hideaki Takagi, Katsuaki Sato, Le Quoc Tuan, Yoshitaka Hishikawa

**Affiliations:** 1 Department of Anatomy, Histochemistry and Cell Biology, Faculty of Medicine, University of Miyazaki, Miyazaki 889–1692, Japan; 2 Faculty of Environment and Natural Resources, Nong Lam University, Ho Chi Minh City, Vietnam; 3 Department of Applied Chemistry, Faculty of Engineering, University of Miyazaki, Miyazaki 889–2192, Japan; 4 Department of Surgery, Faculty of Medicine, University of Miyazaki, Miyazaki 889–1692, Japan; 5 Division of Immunology, Department of Infectious Diseases, Faculty of Medicine, University of Miyazaki, Miyazaki 889–1692, Japan; 6 Present address: Division of Cellular Physiology, Department of Physiology, Faculty of Medicine, Toho University, Tokyo 143–8540, Japan

**Keywords:** photodynamic therapy, phosphorus tetraphenylporphyrin, mitochondrial apoptosis, Bax/Bcl-xL, anticancer effect

## Abstract

Photodynamic therapy (PDT) uses photosensitizer activation by light of a specific wavelength, and is a promising treatment for various cancers; however, the detailed mechanism of PDT remains unclear. Therefore, we investigated the anticancer effect of PDT using a novel phosphorus tetraphenylporphyrin (Ptpp) in combination with light emitting diodes (Ptpp-PDT) in the NOZ human biliary cancer cell line. Cell viability and apoptosis were examined by MTT assay, flow cytometry and TUNEL assay for 24 hr after Ptpp-PDT. MitoTracker and JC-1 were used as markers of mitochondrial localization and membrane potential. The levels of mitochondrial oxidative phosphorylation (OXPHOS) complexes, Bcl-2 family proteins, cytochrome c and cleaved caspase-3 were examined by western blotting and immunohistochemistry. The results revealed that Ptpp localized to mitochondria, and that Ptpp-PDT efficiently decreased cell viability in a dose- and time-dependent manner. JC-1 and OXPHOS complexes decreased, but apoptotic cells increased from 6 to 24 hr after Ptpp-PDT. A decrease in Bcl-xL and increases in Bax, cytochrome c and cleaved caspase-3 were also found from 6 to 24 hr after Ptpp-PDT. Based on these results, we conclude that Ptpp-PDT induces anticancer effects via the mitochondrial apoptotic pathway by altering the Bax/Bcl-xL ratio, and could be an effective treatment for human biliary cancer.

## I Introduction

Photodynamic therapy (PDT) involves administration of a photosensitizing agent followed by activation of the agent by light of a specific wavelength to generate cytotoxic reactive oxygen species that cause cell death [9]. The benefits of PDT include low invasiveness and minimum toxic effects. In addition, PDT does not compromise other treatments and reduces long-term morbidity compared to surgery, chemotherapy or radiotherapy; thus, it can be used for patients with severe conditions and those with no response to conventional therapy [1]. PDT has been shown to be effective as cancer therapy, and may be a promising treatment for incurable cancers, including lung, esophageal, gastric and biliary tract carcinoma [35, 39].

Biliary tract carcinoma, including cholangiocarcinoma and gallbladder cancer, is very aggressive and has a poor prognosis: the 5-year survival rate is 5% to 15% and the median survival time is 12 months [17, 46]. PDT using several photosensitizers, including porfimer sodium, temoporfin and talarporfin sodium, combined with lasers has been used to treat biliary tract carcinoma, but the efficacy is limited [34, 36, 48]. These photosensitizers also have disadvantages of poor water solubility, high skin photosensitivity and low efficiency of singlet oxygen generation [5, 7, 50], while clinical use of lasers is also limited due to cost and complicated maintenance [22, 33]. Therefore, there is a need to develop photosensitizers with high efficiency and minimal side effects that can be used with a low-cost light source.

Use of light emitting diodes (LEDs) of specific wavelengths for PDT may be more convenient and cost-efficient compared to conventional lasers [21, 40]. To improve the efficacy of PDT, we synthesized an amphiphilic phosphorus tetraphenylporphyrin containing axial 3,6-dioxadodecyloxo ligands (Ptpp). This molecule is a novel porphyrin derivative with insertion of a phosphorus atom into the porphyrin ring and modification of the axial ligands with hydrophilic and hydrophobic moieties. Ptpp has higher water solubility and is a more efficient singlet oxygen generator [27, 29]. In addition, PDT using Ptpp in combination with an LED light (Ptpp-PDT) was more effective at a lower concentration than talarporfin sodium in treatment of human biliary cancer cells (NOZ) [30]. However, the precise mechanism of Ptpp-PDT in human cancer cells is largely unknown.

In the current study, the anticancer effects of Ptpp-PDT were examined based on the viability and death of NOZ cells determined by MTT assay, flow cytometry and TUNEL assay. MitoTracker, JC-1, and mitochondrial oxidative phosphorylation (OXPHOS) complexes were used as markers of mitochondrial localization, membrane potential and function, respectively. To investigate apoptosis after Ptpp-PDT, Bcl-2 family proteins, cytochrome c and cleaved caspase-3 were examined by western blotting and immunohistochemistry. Our results showed that Ptpp-PDT induces apoptosis via the mitochondrial pathway by altering the Bax/Bcl-xL ratio in a human biliary cancer cell line.

## II Materials and Methods

### Cell culture

Human biliary cancer cells (NOZ), mouse embryonic fibroblasts (MEF) (Japanese Collection of Research Bioresources, Tokyo, Japan), human breast cancer cells (MCF7), human hepatocyte carcinoma cells (HepG2) and human embryonic kidney 293 cells (HEK293) (Riken Cell Bank, Ibaraki, Japan) were maintained in Dulbecco’s Modified Eagle’s Medium (DMEM) with L-glutamine and phenol red (Fujifilm Wako Pure Chemical Corp., Osaka, Japan) in a humidified atmosphere with 5% CO_2_ at 37°C. The medium was supplemented with 10% (v/v) heat-inactivated fetal bovine serum (Sigma-Aldrich, St. Louis, MO, USA) and penicillin-streptomycin-amphotericin B (Fujifilm Wako).

### PDT experiment

**Fig. 1.**
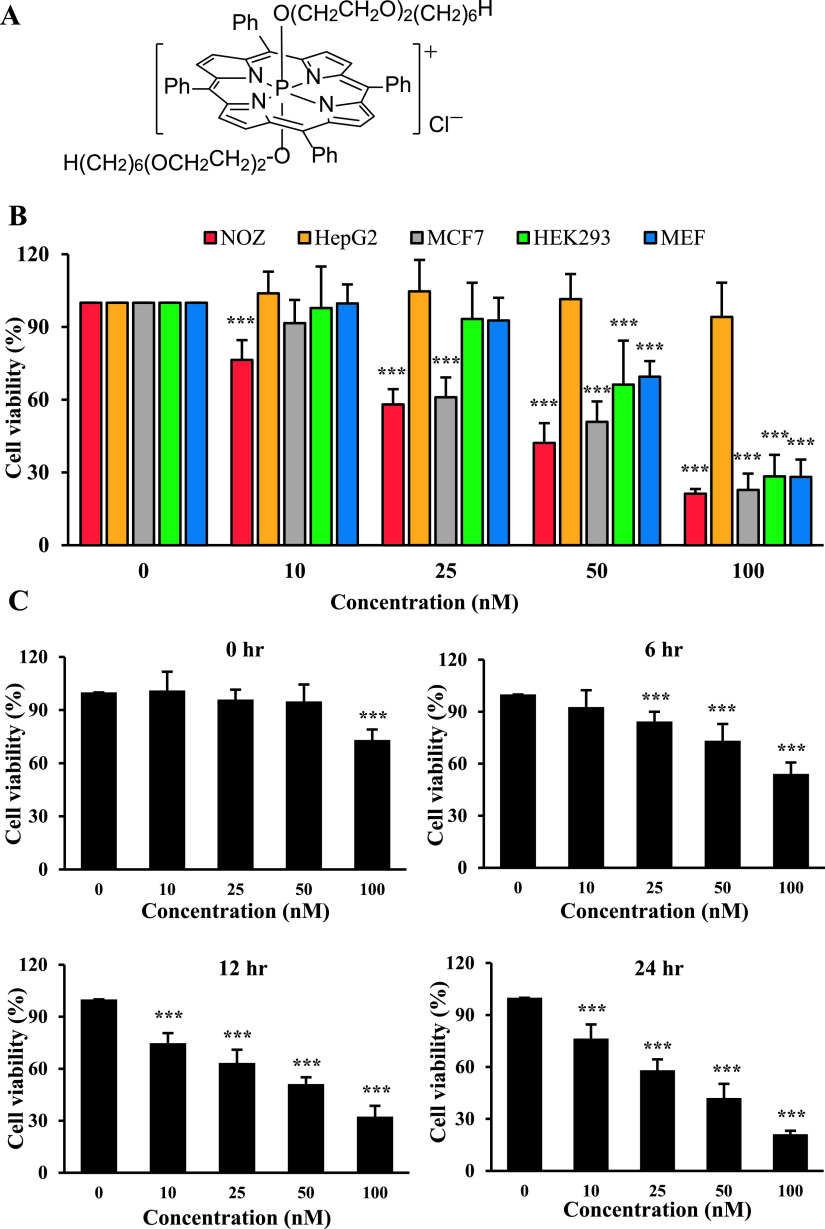
Effect of Ptpp-PDT on cell viability. (**A**) Molecular structure of Ptpp. (**B**) Various cells were incubated with different concentrations of Ptpp (0–100 nM) for 24 hr, and then irradiated with LED for 30 min. After incubation for 24 hr, cell viability was analyzed by MTT assay. (**C**) NOZ cells were treated with different concentrations of Ptpp (0–100 nM) for 24 hr, and then irradiated with LED for 30 min. Cell viability was analyzed by MTT assay at different time points (0–24 hr) after Ptpp-PDT. *P < 0.05, **P < 0.01, ***P < 0.001 vs. 0 nM Ptpp. Data are shown as the mean ± standard deviation (SD) of three independent experiments.

Ptpp was synthesized as reported previously [27, 29] (Fig. 1A). An aqueous stock solution of Ptpp (1 mM) was made by dissolving Ptpp in 10% dimethyl sulfoxide (DMSO) (Fujifilm Wako) in double distilled water and stored at 4°C. DMEM containing Ptpp was prepared from the stock solution before use. Seeded cells were incubated with Ptpp (10, 25, 50 or 100 nM) at 37°C for 24 hr. LED light was irradiated from the bottom of the well plates at 37°C for 30 min (λ = 610 nm, half width = 15 nm, light intensity = 4.09 mW cm^−2^, L610-04, Ushi Opto Semiconductors, Kyoto, Japan). An LED light source was fabricated using 100 plug-in LED arrays on a LED substrate (SPL-100-LC, Revox, Kanagawa, Japan). A heat-adsorbing filter filled with water (100 mm in diameter and 20 mm in thickness) was placed between the well plate and the LED light source to minimize heat emitted from the LED [30].

### MTT assay

Cell viability was examined by 3-(4,5-dimethyl-2-thiazolyl)-2,5-diphenyltetrazolium bromide (MTT) assay [49]. Briefly, cells were seeded in a 96-well plate at a density of 2 × 10^3^ cells/well and incubated at 37°C overnight. After PDT using Ptpp at concentrations of 10, 25, 50 or 100 nM, cells were incubated at 37°C for 0, 6, 12 or 24 hr. After adding MTT solution (5 mg/ml) (Nacalai Tesque, Kyoto, Japan), the cells were incubated at 37°C for 2 hr. After removal of the MTT reagent, formazan crystals were dissolved in DMSO. The resulting intracellular purple formazan was quantified with a spectrophotometer at an absorbance of 562 nm using Immuno Mini NJ-2300 (Nalge Nunc Int. Co. Ltd., Tokyo, Japan).

### Ptpp absorbance measurement

NOZ and HepG2 cells were seeded in 3-cm dishes at a density of 2 × 10^5^ cells/dish and incubated at 37°C overnight. Subsequently, the cells were incubated with 1 μM Ptpp for 1, 4, 8 or 24 hr. Cell lysates were then prepared in 5% sodium dodecyl sulfate (SDS) buffer (Fujifilm Wako) and Ptpp absorbance was measured at 430 nm using a UV-Vis spectrometer (V-550; Jasco Co., Tokyo, Japan) [30].

### Localization of Ptpp

To examine the localization of Ptpp, NOZ and HepG2 cells were seeded onto coverslips in 12-well plates at a density of 1 × 10^5^ cells/well and incubated at 37°C overnight. Subsequently, the cells were incubated with 50 nM Ptpp for 24 hr, and then washed with phosphate-buffered saline (PBS; pH 7.4, 0.01 M) and fixed with 4% paraformaldehyde (PFA) (Merck, Darmstadt, Germany) in PBS for 15 min at room temperature (RT). For detection of colocalization with mitochondria, after incubation with 50 nM Ptpp for 24 hr, NOZ cells were washed with PBS and incubated with 200 nM MitoTracker Green^®^ (Thermo Fisher Scientific, Waltham, MA, USA) at 37°C for 30 min. The cells were then washed with PBS and fixed with 4% PFA in PBS at RT for 15 min. Nuclei were counterstained with 4',6-diamidino-2-phenylindole (DAPI) (Thermo Fisher Scientific). Images were captured with a Zeiss LSM700 confocal microscope (Carl Zeiss AG, Oberkochen, Germany).

### Flow cytometry

NOZ cells were seeded in a 3-cm dish at a density of 2 × 10^5^ cells/dish and incubated at 37°C overnight. Cells treated with 50 nM Ptpp only were defined as the 0 hr time point. For evaluation of the mitochondrial membrane potential, after PDT using 50 nM Ptpp for 24 hr, NOZ cells were harvested and incubated with 4 μM 5,5',6,6'-tetrachloro-1,1',3,3'-tetramethylbenzimidazolylcarbocyanine iodide (JC-1) (Dojindo Molecular Technologies, Kumamoto, Japan) at 37°C for 30 min. For detection of apoptosis, NOZ cells were harvested 1 to 24 hr after PDT and incubated with Annexin V-FITC and propidium iodide (PI) (Nacalai Tesque) at RT for 15 min [26]. The mitochondrial membrane potential and apoptotic cells were analyzed by flow cytometry using FACSCalibur (BD Biosciences, San Jose, CA, USA).

### Western blotting

Total NOZ cell lysates were prepared using hot SDS buffer containing 0.9% SDS, 15 mM ethylenediaminetetraacetic acid (EDTA), 8 mM unlabeled methionine and a protease inhibitor cocktail. Lysates were boiled for 10 min, cooled and diluted in 0.3% SDS, then adjusted to contain 33 mM Tris/acetate, pH 8.5, and 1.7% Triton X-100 [13]. The protein concentration was determined using a Pierce BCA protein assay kit (Thermo Fisher Scientific). Equal amounts of protein were mixed with loading buffer (0.2 M Tris-HCl, pH 8.0, 0.5 M sucrose, 5 mM EDTA, 0.01% bromophenol blue, 10% 2-mercaptoethanol, and 2.5% SDS), boiled for 5 min, separated by SDS-polyacrylamide gel electrophoresis (SDS-PAGE), and transferred onto polyvinylidene difluoride membranes (Bio-Rad, Hercules, CA, USA). The membranes were blocked with 5% nonfat skim milk in Tris-buffered saline with 0.1% Tween 20 (TBST; 20 mM Tris buffer, pH 7.6, and 150 mM NaCl) for 1 hr at RT, and then incubated overnight with primary antibodies against OXPHOS I (sc-515527), Bcl-2 (sc-492, Santa Cruz Biotechnology, Dallas, Texas, USA), OXPHOS III (ab14745), OXPHOS V (ab110273, Abcam, Cambridge, UK), Bak (#12105), Bok (#86875), Bim (#2933), Bad (#9239), Puma (#12450), Noxa (#14766), Bid (#2002), Bik (#4592, 1:1000), and cleaved caspase-3 (#9664, 1:500, Cell Signaling Technology, Danvers, MA, USA) or β-actin (A3854, 1:400,000, Sigma-Aldrich). The membranes were washed with TBST and incubated with horseradish peroxidase (HRP)-conjugated goat anti-rabbit or mouse IgG (1:5000, Dako, Glostrup, Denmark) at RT for 1 hr. Protein bands were detected using EzWestLumi plus (ATTO Corp., Tokyo, Japan), captured with LAS4000 (Fujifilm, Tokyo, Japan), and analyzed by ImageJ (ver.1.51n, NIH Software, Bethesda, MD, USA).

### Immunohistochemistry

Immunohistochemistry was performed as reported previously [2, 6]. Cells were seeded on coverslips in 12-well plates at a density of 1 × 10^5^ cells/well and incubated at 37°C overnight. NOZ cells treated with 50 nM Ptpp only were defined as the 0 hr time point. After PDT using 50 nM Ptpp for 1 to 24 hr, cells were washed with PBS, fixed with 4% PFA in PBS for 15 min and permeabilized with 0.2% Triton X-100 in PBS at RT for 10 min. Cellular endogenous peroxidase activity was blocked with 0.3% H_2_O_2_ (Fujifilm Wako) in methanol for 30 min and nonspecific binding sites were blocked by incubation with 500 μg/ml normal goat IgG (I9140) in 1% bovine serum albumin (Sigma-Aldrich) in PBS for 30 min. Cells were incubated with Bcl-2 (1:200), Bcl-xL (sc-8392, 1:200), cytochrome c (sc-13156, 1:400, Santa Cruz Biotechnology), Bax (MA5-14003, 1:50, Thermo Fisher Scientific), or cleaved caspase-3 (1:100) for 2 hr and washed in 0.075% Brij L23 (Sigma-Aldrich) in PBS. The cells were then incubated with HRP-conjugated goat anti-rabbit or mouse IgG for 1 hr and washed with 0.075% Brij L23 in PBS. After visualization with 3-amino-9-ethylcarbazole (Nichirei Biosciences, Tokyo, Japan), nuclei were counterstained with methyl green (Sigma-Aldrich). As a negative control, normal rabbit or mouse IgG (Dako) was used at the same concentration as the primary antibody in each experiment. Microphotos were captured with an Olympus microscope (BX53, Tokyo, Japan). For quantitative analysis of Bcl-xL, Bax and cleaved caspase-3, at least 1000 cells were counted in random fields, and results are shown as the percentage of positive cells per total number of counted cells.

### TUNEL assay

Apoptotic cells were detected with a MEBSTAIN Apoptosis TUNEL Kit Direct (8445, Medical and Biological Laboratories, Nagoya, Japan) [26]. Briefly, cells were fixed with 4% PFA in PBS and permeabilized with 0.2% Triton X-100 in PBS. The TUNEL reaction was performed for 1 hr at 37°C. Nuclei were counterstained with DAPI. Images were captured with a Zeiss LSM700 confocal microscope.

### Statistical analysis

All analyses were performed with Statistical Package for Social Sciences (SPSS ver.20, Chicago, IL, USA) software. Data are expressed as means ± standard deviation (SD) from three independent experiments. Differences between experimental groups were assessed by Student’s *t*-test, with *P* < 0.05 considered to be significant.

## III Results

### Ptpp-PDT strongly decreases the viability of NOZ cells

The effect of Ptpp-PDT on cell viability was examined by MTT assay in various cell lines. Ptpp-PDT decreased the viability of NOZ, MCF7, HEK293 and MEF cells in a dose-dependent manner, but did not affect the viability of HepG2 cells. In particular, NOZ cells were more sensitive to Ptpp-PDT than other cell lines (Fig. 1B). At 10 nM Ptpp-PDT, only NOZ cells were significantly decreased, but at 100 nM there were no significant differences among NOZ, MCF7, HEK293 and MEF cells. Ptpp-PDT significantly decreased the viability of NOZ cells in a time- and dose-dependent manner (Fig. 1C). The half-maximal inhibitory concentration (IC_50_) of Ptpp was 35.5 nM at 24 hr after Ptpp-PDT, and PDT using 50 nM Ptpp decreased the cell viability by 57.9 ± 8.2%. These results show that Ptpp-PDT efficiently decreases the viability of NOZ cells. Based on these results, 50 nM Ptpp was selected as the concentration for subsequent experiments on the effects of Ptpp-PDT on NOZ cells.

### Ptpp localizes to mitochondria

**Fig. 2.**
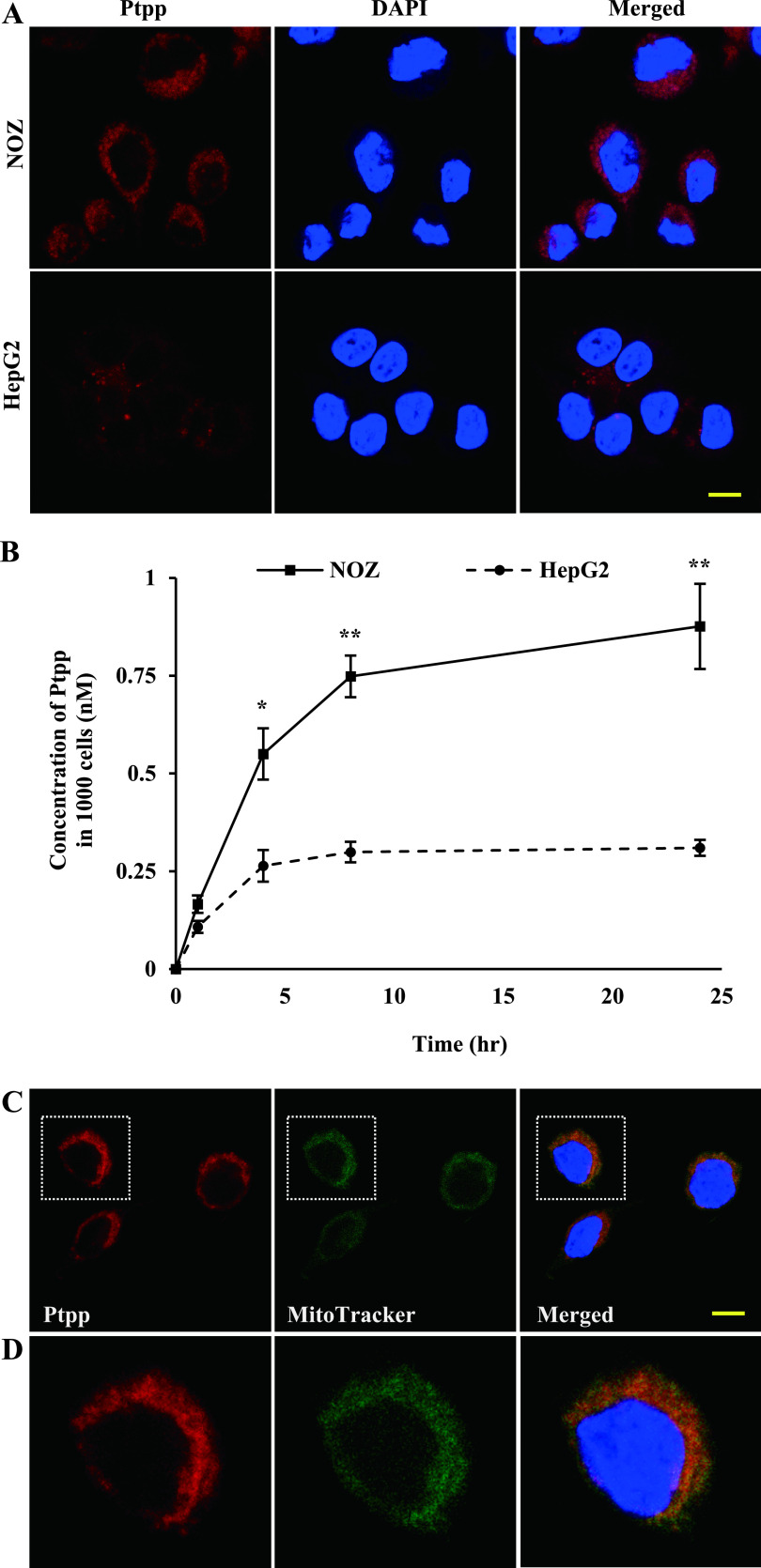
Uptake of Ptpp in NOZ and HepG2 cells, and subcellular localization of Ptpp in NOZ cells. (**A**) Fluorescence of Ptpp in NOZ and HepG2 cells. Cells were incubated with 50 nM Ptpp for 24 hr. Red and blue colors represent Ptpp and DAPI, respectively. Bar = 10 μm. (**B**) Quantitative analysis of Ptpp absorbance. Cells were incubated with 1 μM Ptpp for 1, 4, 8 or 24 hr. The results in the line graph are Ptpp concentrations in 1000 cells. *P < 0.05, **P < 0.01 vs. HepG2 cells at each time point. Data are shown as the mean ± SD of three independent experiments. (**C**) Low- and (**D**) high-magnification micrographs of subcellular localization of Ptpp in NOZ cells. Cells were incubated with 50 nM Ptpp for 24 hr and 200 nM MitoTracker Green^®^ for 30 min. Red, green and blue colors represent Ptpp, MitoTracker and DAPI, respectively. Bar = 10 μm.

Cellular uptake and accumulation of Ptpp may influence the efficiency of PDT. Therefore, we examined the subcellular localization of Ptpp in NOZ and HepG2 cells, which were the most and least sensitive cell lines, respectively, in the MTT assay. Ptpp localized to the cytoplasm and was much more abundant in NOZ cells than in HepG2 cells (Fig. 2A). To confirm these results, intracellular Ptpp was quantified in NOZ and HepG2 cell lysates (Fig. 2B). NOZ cells contained three times as much Ptpp as HepG2 cells, and this result was corroborated by confocal microscopy findings. Ptpp accumulation also correlated strongly with cell viability (Fig. 1B). Localization of photosensitizers plays an important role in the mechanism of cell death after PDT [23, 32]. Thus, the subcellular localization of Ptpp in NOZ cells was determined by confocal microscopy, which revealed that Ptpp colocalized with mitochondria (Fig. 2C, D).

### Ptpp-PDT induces mitochondrial dysfunction in NOZ cells

**Fig. 3.**
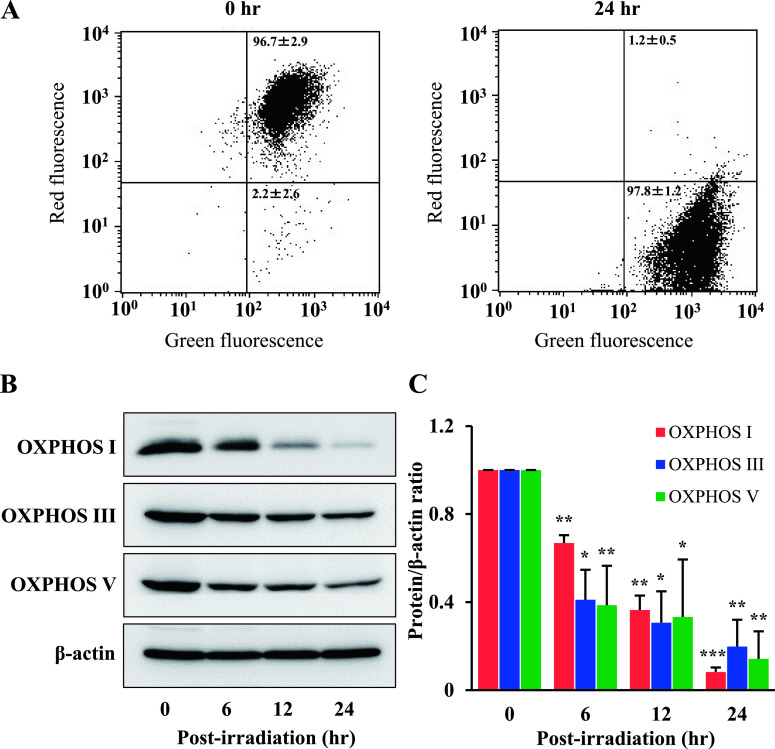
Effect of Ptpp-PDT on mitochondrial functions in NOZ cells. (**A**) The mitochondrial membrane potential was analyzed by flow cytometry using 4 μM JC-1 at 0 and 24 hr after Ptpp-PDT. (**B**) Western blot analysis of OXPHOS I (23 kDa), III (48 kDa) and V (55 kDa) in NOZ cells at 0, 6, 12 and 24 hr after Ptpp-PDT. Protein extracts (10 μg) were subjected to SDS-PAGE. (**C**) Protein bands were analyzed by densitometry and results are shown as a bar graph. Protein expression was normalized to β-actin (42 kDa). *P < 0.05, **P < 0.01, ***P < 0.001 vs. 0 hr for each protein. Data are shown as the mean ± SD of three independent experiments.

To assess the effect of Ptpp-PDT on mitochondrial functions, the mitochondrial membrane potential was measured by flow cytometry using JC-1 staining. At 0 hr, red emission from JC-1 aggregates was dominant, indicating that the membrane potential was preserved. However, JC-1 emission shifted from red to green after Ptpp-PDT, indicating a decrease in membrane potential and depolarization of mitochondria at 24 hr (Fig. 3A). In evaluation of OXPHOS complexes by western blotting (Fig. 3B), Ptpp-PDT significantly decreased the levels of OXPHOS I, III and V by 1.5, 2.4 and 2.6 times at 6 hr, and by 12.2, 5.1 and 7 times at 24 hr after Ptpp-PDT, respectively (Fig. 3C). These results indicate changes to the OXPHOS system after Ptpp-PDT in NOZ cells.

### Ptpp-PDT increases apoptotic cell death in NOZ cells

**Fig. 4.**
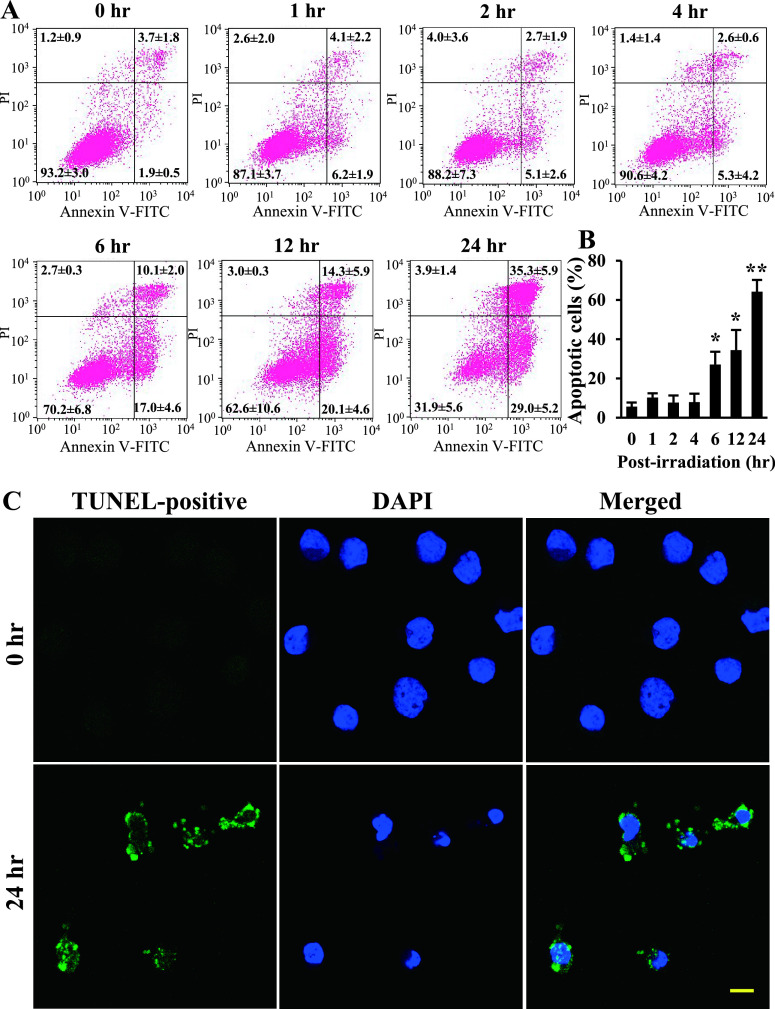
Effect of Ptpp-PDT on induction of apoptotic cell death in NOZ cells. (**A**) Apoptosis and necrosis were analyzed by flow cytometry using annexin V-FITC and PI at different time points (0–24 hr) after Ptpp-PDT. (**B**) Data were obtained from three independent experiments and results are shown as a bar graph. Annexin V-positive/PI-positive and -negative cells are apoptotic, and annexin V-negative/PI-positive cells are necrotic. *P < 0.05, **P < 0.01 vs. 0 hr. Data are shown as the mean ± SD of three independent experiments. (**C**) Apoptotic cells were examined by TUNEL assay at 0 and 24 hr after Ptpp-PDT. Green and blue colors represent TUNEL-positive cells and DAPI, respectively. Bar = 10 μm.

Functional alterations of mitochondria often induce cell death. Therefore, flow cytometry was performed to detect apoptotic and necrotic cells after Ptpp-PDT (Fig. 4A). The number of apoptotic cells was significantly increased to 27.1 ± 6.6, 34.4 ± 10.3 and 64.2 ± 6.0% at 6, 12 and 24 hr after Ptpp-PDT, respectively (Fig. 4B). In contrast, there were no differences in the number of necrotic cells at any time point. Apoptosis was confirmed in a TUNEL assay (Fig. 4C), which showed a marked increase in the number of TUNEL-positive cells at 24 hr after Ptpp-PDT, compared to that at 0 hr.

### Ptpp-PDT induces the mitochondria-dependent apoptosis pathway in NOZ cells

**Fig. 5.**
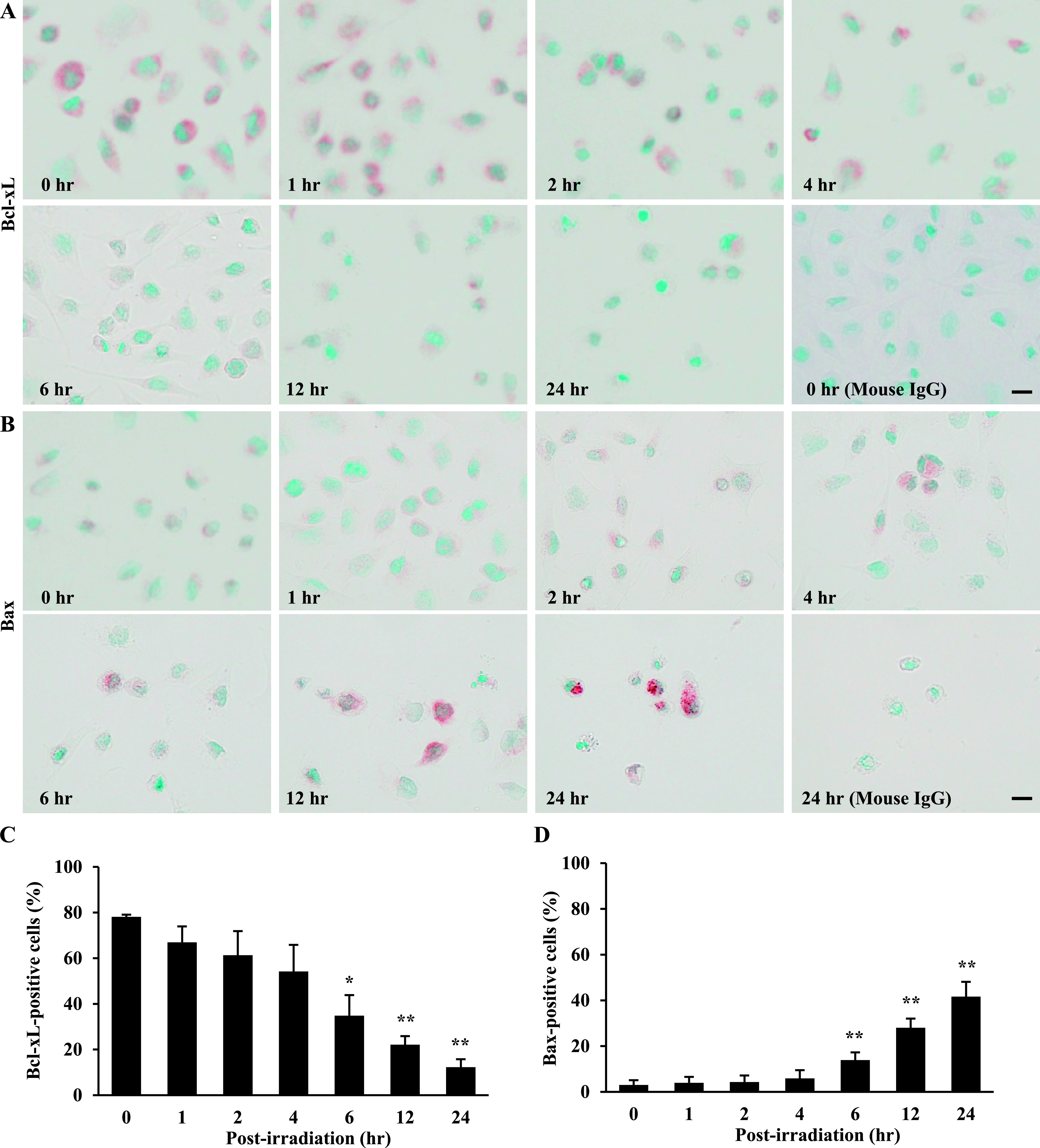
Effect of Ptpp-PDT on Bcl-xL and Bax in NOZ cells. Localization of (**A**) Bcl-xL and (**B**) Bax determined immunohistochemically at different time points (0–24 hr) after Ptpp-PDT. Quantitative analysis of (**C**) Bcl-xL-positive and (**D**) Bax-positive cells. *P < 0.05, **P < 0.01 vs. 0 hr. Data are shown as the mean ± SD of three independent experiments. Bar = 10 μm.

To investigate the apoptotic pathway in Ptpp-PDT, we examined expression of Bcl-2 family proteins in NOZ cells treated with Ptpp-PDT, using western blotting and immunohistochemistry. Interestingly, Bcl-xL was strongly expressed at 0 hr; however, the number of Bcl-xL-positive cells was significantly decreased to 34.8 ± 9.0, 22.1 ± 3.8 and 12.3 ± 3.5% at 6, 12 and 24 hr after Ptpp-PDT, respectively (Fig. 5A, C). In contrast, Bax expression was significantly increased to 13.9 ± 3.4, 28.0 ± 4.1 and 41.7 ± 6.4% at 6, 12 and 24 hr after Ptpp-PDT, respectively (Fig. 5B, D). In normal conditions, Bax formed a speckled pattern, whereas after Ptpp-PDT, strong Bax staining occurred over the whole cytoplasm. Anti-apoptotic protein Bcl-2 and pro-apoptotic Bak, Noxa and Bid were also detected, but showed no changes in expression after Ptpp-PDT (data not shown). Pro-apoptotic proteins, such as Bok, Bim, Bad, Bik and Puma, were not detected in NOZ cells (data not shown).

**Fig. 6.**
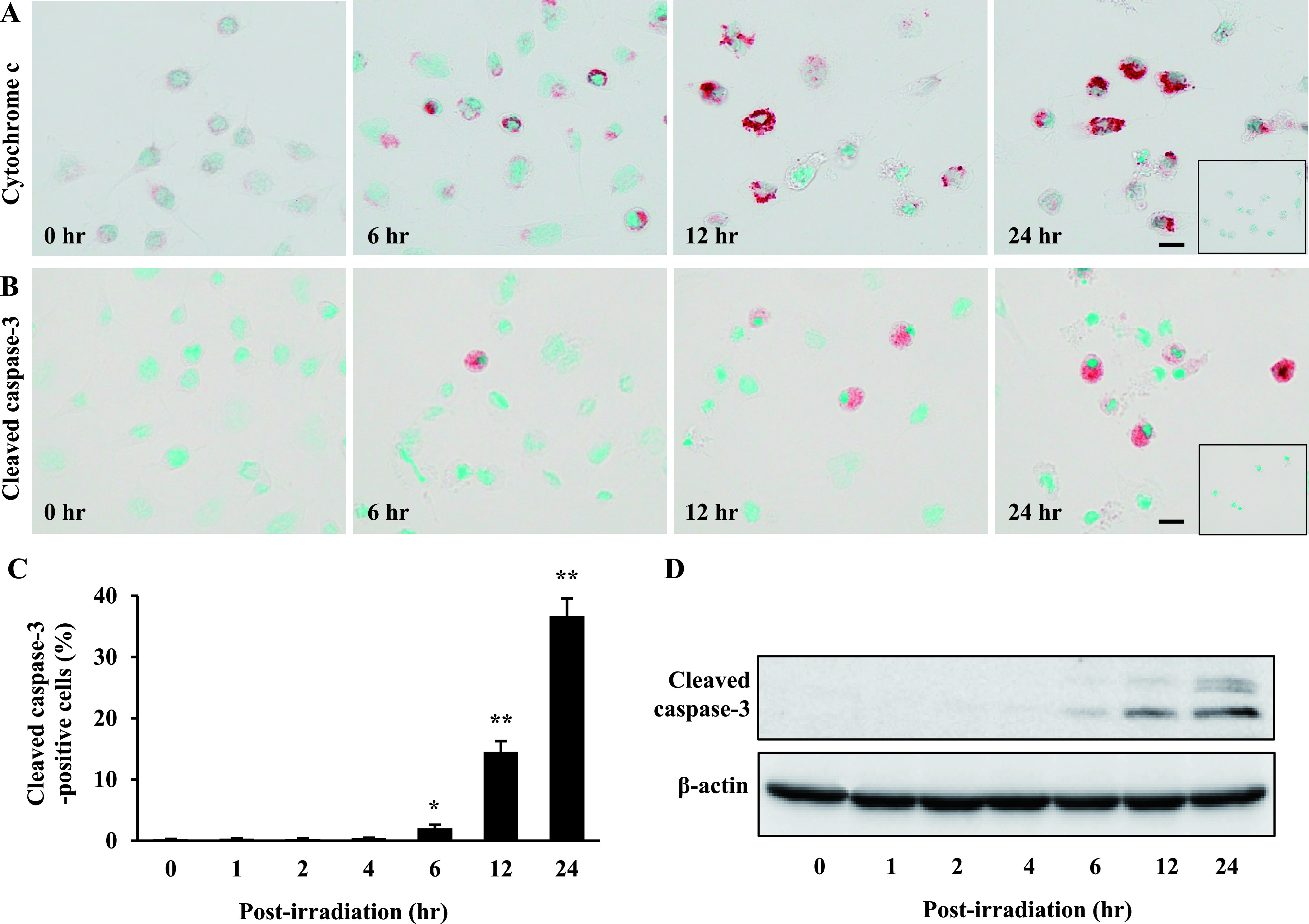
Effect of Ptpp-PDT on cytochrome c and cleaved caspase-3 in NOZ cells. Immunohistochemical localization of (**A**) cytochrome c and (**B**) cleaved caspase-3 at 0, 6, 12 and 24 hr after Ptpp-PDT. Negative controls are shown in the inset. Bar = 10 μm. (**C**) Quantitative analysis of cleaved caspase-3-positive cells. *P < 0.05, **P < 0.01 vs. 0 hr. Data are shown as the mean ± SD of three independent experiments. (**D**) Western blot analysis detected double bands for cleaved caspase-3 (17 and 19 kDa). Protein extracts (20 μg) were subjected to SDS-PAGE. β-actin (42 kDa) was used as a loading control.

A shift in expression of Bcl-xL and Bax leads to release of cytochrome c into the cytosol and further activates caspase-3 [41, 43, 44]. In our findings, cytochrome c was markedly increased at 6, 12 and 24 hr after Ptpp-PDT, compared to cells at 0 hr (Fig. 6A). Consistent with this, there was an increase in cleaved caspase-3-positive cells at 6, 12 and 24 hr after Ptpp-PDT based on immunohistochemistry and western blotting (Fig. 6B, D). In particular, the number of cleaved caspase-3-positive cells was 2.0 ± 0.6 to 36.7 ± 2.9% at 6 to 24 hr, whereas that number was less than 0.4 ± 0.1% at 0 to 4 hr after Ptpp-PDT (Fig. 6C).

## IV Discussion

In this study, we investigated the anticancer mechanism of Ptpp-PDT in NOZ cells. The results showed that Ptpp, a novel porphyrin, localized to mitochondria and caused an imbalance in expression of Bcl-xL and Bax that induced mitochondria-related apoptosis. Thus, Bcl-xL and Bax may be important for the effect of PDT in human biliary cancer.

Apoptosis is thought to be a prominent cell death mechanism in cells responding to PDT [1, 39]. Similarly, our results revealed that the number of apoptotic, but not necrotic, NOZ cells significantly increased after Ptpp-PDT. These findings indicate that the decrease in cell viability after Ptpp-PDT occurred mainly through apoptosis. Members of the Bcl-2 family are key regulators of mitochondria-related apoptosis [3, 8], and an imbalance in expression of anti-apoptotic Bcl-xL or Bcl-2 and pro-apoptotic Bax leads to apoptosis; therefore, the Bax/Bcl-xL or Bax/Bcl-2 ratio is an important indicator of apoptosis [3, 15, 43, 44]. Many studies have shown that the Bax/Bcl-2 ratio is the main factor for apoptosis induction in cancers such as breast, prostate, liver and lung cancer [4, 16, 18, 42]. The Bax/Bcl-xL ratio is also associated with induction of apoptosis in several cancer cell lines, such as pancreatic neoplasia and mesothelioma cells [19, 31]. Thus, regulation of mitochondrial apoptosis may depend on different expression patterns of Bcl-2 family proteins in different types of cancers.

In the current study, Bcl-xL expression decreased and Bax expression gradually increased after Ptpp-PDT, whereas Bcl-2, Bak, Noxa and Bid were unchanged. Increased levels of cytochrome c and activated caspase-3 were also observed with an increase in TUNEL-positive cells after Ptpp-PDT. Therefore, Bcl-xL, but not Bcl-2, plays a crucial role in the mitochondrial apoptotic pathway after Ptpp-PDT in NOZ cells. The mechanism of Ptpp-PDT differs from that of PDT using porfimer sodium, temoporfin and talaporfin sodium photosensitizers. Porfimer sodium mainly induces necrosis, endoplasmic reticulum-localized temoporfin induces photoactivation of the caspase-7 apoptotic pathway, and lysosomal-localized talaporfin sodium causes apoptosis through leakage of cathepsins and Bid cleavage [1, 38, 39]. Since the anticancer mechanism of PDT varies depending on the photosensitizer, more effective cancer treatment may be achieved using a combination of these agents.

Our results revealed that Ptpp-PDT strongly decreased the viability of NOZ cells in a dose-dependent manner, but did not affect the viability of HepG2 cells. These results correlated strongly with accumulation of Ptpp in NOZ and HepG2 cells. Other studies have similarly shown that the efficacy of PDT with various photosensitizers has a clear correlation with their fluorescence intensity [11, 25]. Overexpression of adenosine triphosphate-binding cassette (ABC) efflux transporters, such as ABCB1 (multidrug resistance protein 1 (MDR1)/permeability glycoprotein (P-gp)), ABCC1 (multidrug resistance-associated protein 1 (MRP1)) and breast cancer resistance protein ABCG2 (BCRP), is a key factor in PDT resistance in colon adenocarcinoma and lung carcinoma cells [14, 20, 51]. P-gp and BCRP are highly expressed in HepG2 cells [10, 24], which may be related to lower accumulation of Ptpp in these cells compared to NOZ cells.

Singlet oxygen generated by PDT has a short lifetime and limited diffusion in cells, indicating that the resulting photodynamic damage occurs close to the intracellular location of the photosensitizer [1, 45]. Thus, localization of the photosensitizer is a crucial parameter that influences the extent of cell damage, as well as the mechanism of cell death [23, 32]. Depending on the subcellular localization of photosensitizers, PDT-induced cell death involves plasma membrane death receptors, mitochondria, lysosomes or endoplasmic reticulum pathways [39]. We found that Ptpp was taken up into the cytoplasm and localized to mitochondria, which are key players in generation of cellular adenosine triphosphate by the OXPHOS system, which consists of five protein complexes. Targeting or inhibition of any of these complexes results in cell death [12, 41], and decreased expression of OXPHOS I, III and V in NOZ cells occurred after Ptpp-PDT. Similarly, PDT with mitochondria-targeted photosensitizers has been shown to enhance mitochondrial damage, including disruption of the transmembrane potential and inhibition of the OXPHOS system [47]. Moreover, such photosensitizers are particularly efficient for PDT, possibly because they induce apoptosis directly [39]. Taken together, these results suggest that Ptpp-PDT may directly disrupt mitochondrial functions, leading to induction of cell death in NOZ cells.

In the clinical setting, PDT for biliary tract cancer is currently performed using porfimer sodium or talarporfin sodium as a photosensitizer [33, 37]. Porfimer sodium is effective at a high dose, but patients experience prolonged skin photosensitivity as a side effect [38]. In contrast, PDT using talarporfin sodium has less skin phototoxicity, but its clinical efficacy for biliary tract carcinoma has yet to be shown [35]. For development of a new photosensitizer with higher efficiency and lower phototoxicity, we synthesized Ptpp through modification of the axial ligands with hydrophilic and hydrophobic moieties, which prevents formation of porphyrin-ring aggregates and improves incorporation of Ptpp into the target cell [28]. Thus, Ptpp-PDT is more effective at a lower concentration (IC_50_ = 35.5 nM) in NOZ cells compared to talarporfin sodium-PDT (IC_50_ = 7570 nM) [30]. The efficacy of Ptpp at the nanomolar level may help to reduce prolonged skin photosensitivity. Therefore, our findings may be helpful for development of an efficient photosensitizer for PDT with minimal side effects.

In conclusion, Ptpp-PDT may induce apoptosis through the mitochondrial pathway by altering the Bax/Bcl-xL ratio in NOZ cells, and Ptpp may be an effective photosensitizer in PDT for biliary cancer.

## V Conflicts of Interest

The authors declare that there are no conflicts of interest.

## VI Acknowledgments

This study was supported in part by Grants-in-Aid for Scientific Research from the Japan Society for the Promotion of Science (No. 16K08471 to Y. Hishikawa, No. 16K05847 to J. Matsumoto, No. 19K09178 to A. Nanashima).
